# Supplementary material for: Genetic labeling of embryonically-born dentate granule neurons in young mice using the PenkCre mouse line
Source: Sci Rep. 2024 Feb 29;14:5022. doi: 10.1038/s41598-024-55299-9 (PMC10904803; doi:10.1038/s41598-024-55299-9)
Supplement: Supplementary file 1 — Supplementary Figures. [file 41598_2024_55299_MOESM1_ESM.pdf]

SUPPLEMENTARY INFORMATION

Genetic labeling of embryonically-born dentate granule neurons in young mice using the *Penk*^{Cre} mouse line

Pierre Mortessagne [1,3], Estelle Cartier [1,3], Maddalena Balia [2], Murielle Fèvre [1], Fiona Corailler [1], Cyril Herry [1], Djoher Nora Abrous [1 *], Arne Battefeld [2], Emilie Pacary [1 *]

SUPPLEMENTARY FIGURE LEGENDS

Figure S1: Repartition of Penk^{Cre}+ cells in the mouse brain.

(A) Penk^{Cre}+ cells along the septo-temporal axis (coordinates from the Bregma in white) in the *Penk^{Cre};Ai6* mouse brain at P35. One series of 40 µm sections with 400 µm between two successive sections. (B) Illustration of Penk^{Cre}+ neurons in the hilus of a 1-month-old *Penk^{Cre};Ai6* mouse that express the interneuron markers VIP or calretinin. White arrowheads indicate double positive cells. GCL: granule cell layer, ML: molecular layer.

Scale bars represent 2.5 mm (A), 20 µm (B).

Figure S2: Birthdating of PenkCre+ cells in the two blades of the DG.

Quantification of the percentage of ZsGreen+/EdU cells over total ZsGreen+ cells in the (A) supra and (B) infra-pyramidal blades at P35 and after injection of EdU at different time points during pregnancy, n = 3 mice per time point from at least two different litters.

Figure S3: Contributions of principal components and synaptic properties of DGNs in *Penk^{Cre};Ai6* mice

(A) Oblique contrast image of DGNs in the suprapyramidal blade from an acute horizontal slice. A whole-cell patch-clamp pipette can be seen in contact with a ZsGreen– neuron (arrow). (B) Examples of voltage-clamp recordings from ZsGreen+ and ZsGreen– DGNs showing post synaptic currents (PSC) in the absence of pharmacological isolation. (C) (D) Boxplots of PSC frequency (Mann-Whitney test) and PSC amplitude (t-test) from ZsGreen+ and ZsGreen– neurons show no difference in post-synaptic properties. (E) Plot showing the contribution of the selected morpho-electric parameters to the first two principal components.

Figure S4: Temporal DG of *Penk^{Cre}; Ai6* mice at 1, 3 and 6 months

Scale represents 50 µm.

Figure S5: *Penk* is progressively expressed from the outer GCL to the inner GCL with age.

(A-D) DG of *Penk*^{Cre}*;Ai14* mice in (A, C) 1 month-old and (B, D) 1 year-old animals. (C, D) Higher magnifications were acquired from different sections and the white box is indicative of the location. (E) *Penk* in situ hybridization in mouse sagittal sections from the Allen Brain Atlas.

Scale bars represent 100 µm (A, B, E) and 20 µm (C, D).

Figure S1

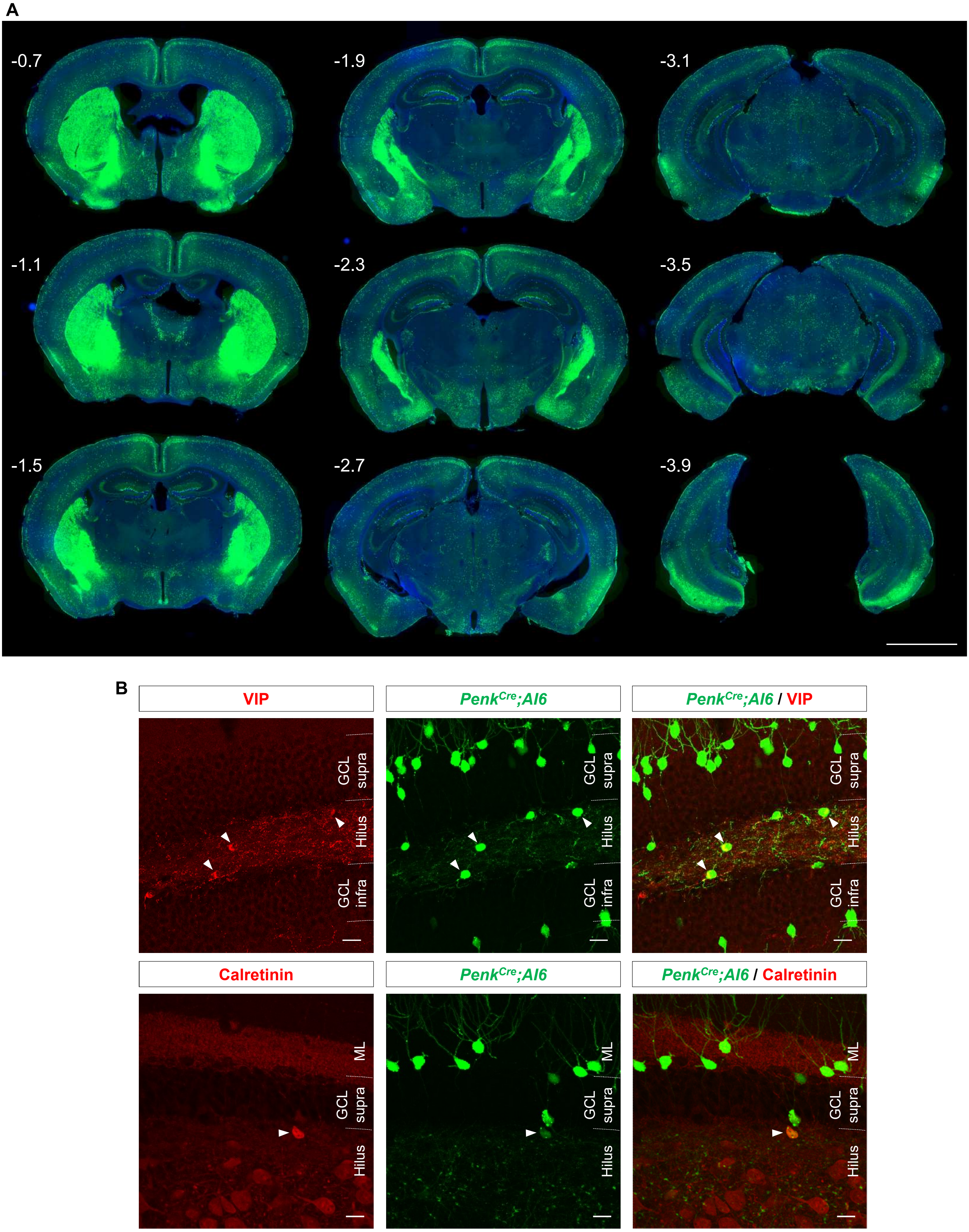
A
-0.7
-1.9
-3.1
-1.1
-2.3
-3.5
-1.5
-2.7
-3.9
B
VIP
PenkCre;Al6
PenkCre;Al6 / VIP
GCL supra
Hilus
GCL infra
Calretinin
PenkCre;Al6
PenkCre;Al6 / Calretinin
ML
GCL supra
Hilus

Figure S2

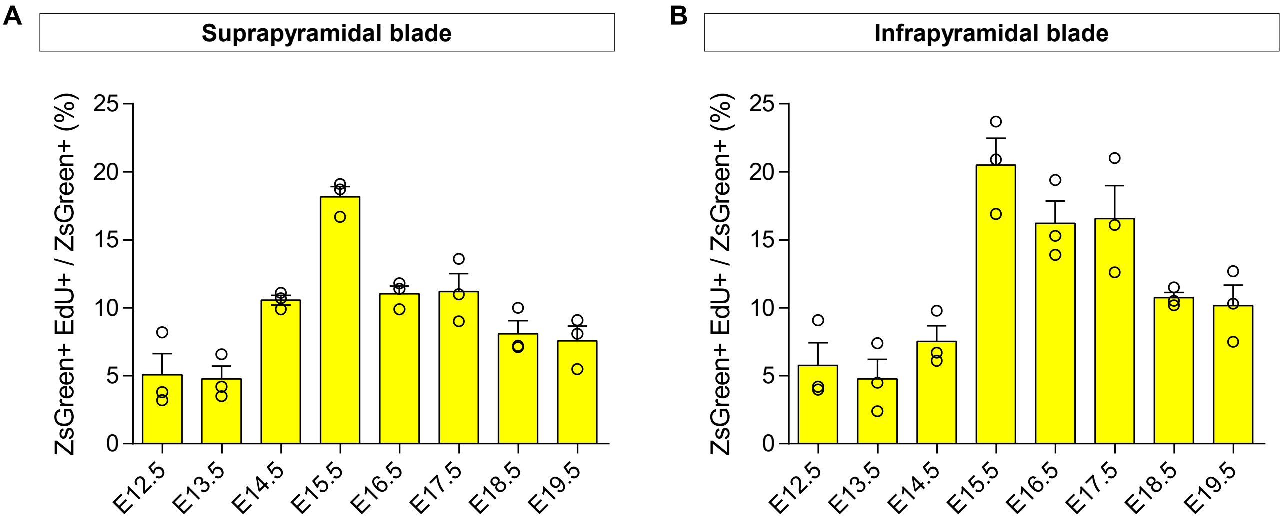
A
Suprapyramidal blade
ZsGreen+ EdU+ / ZsGreen+ (%)
0
5
10
15
20
25
E12.5
E13.5
E14.5
E15.5
E16.5
E17.5
E18.5
E19.5
B
Infrapyramidal blade
ZsGreen+ EdU+ / ZsGreen+ (%)
0
5
10
15
20
25
E12.5
E13.5
E14.5
E15.5
E16.5
E17.5
E18.5
E19.5

Figure S3

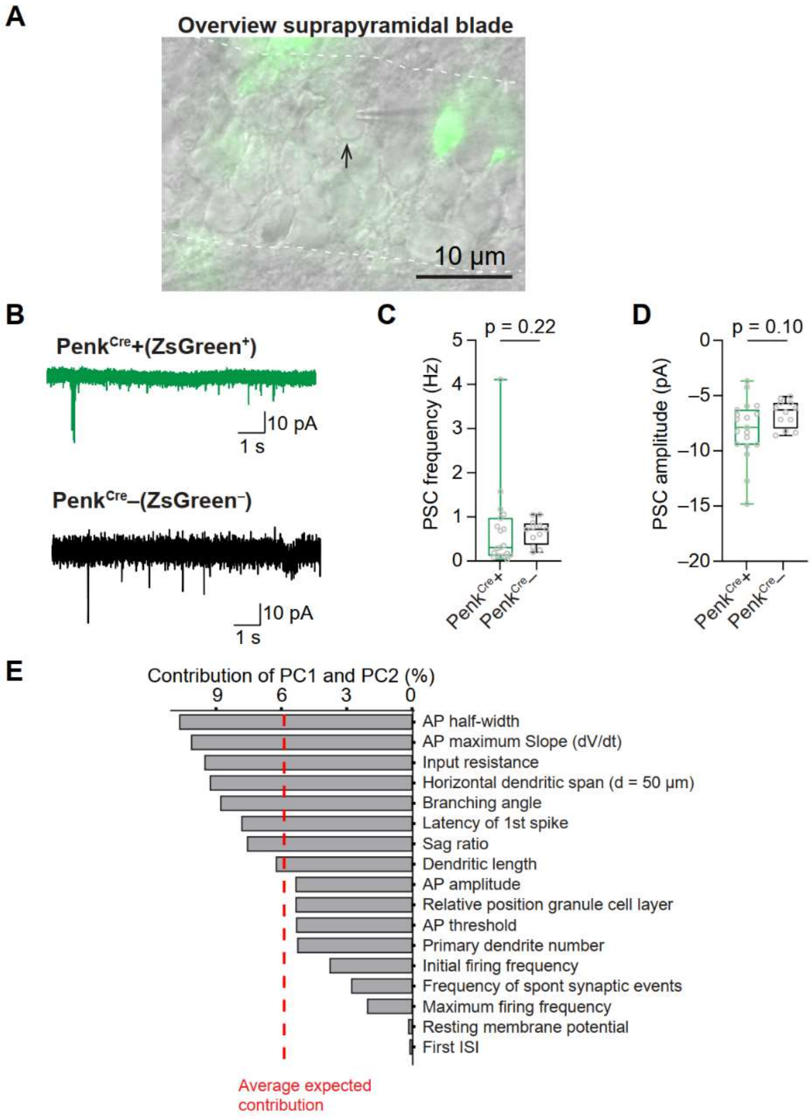
A
Overview suprapyramidal blade
10 µm
B
Penk^Cre+(ZsGreen+)
10 pA
1 s
Penk^Cre–(ZsGreen–)
10 pA
1 s
C
p = 0.22
PSC frequency (Hz)
Penk^Cre+
Penk^Cre–
D
p = 0.10
PSC amplitude (pA)
Penk^Cre+
Penk^Cre–
E
Contribution of PC1 and PC2 (%)
AP half-width
AP maximum Slope (dV/dt)
Input resistance
Horizontal dendritic span (d = 50 µm)
Branching angle
Latency of 1st spike
Sag ratio
Dendritic length
AP amplitude
Relative position granule cell layer
AP threshold
Primary dendrite number
Initial firing frequency
Frequency of spont synaptic events
Maximum firing frequency
Resting membrane potential
First ISI
Average expected contribution

Figure S4

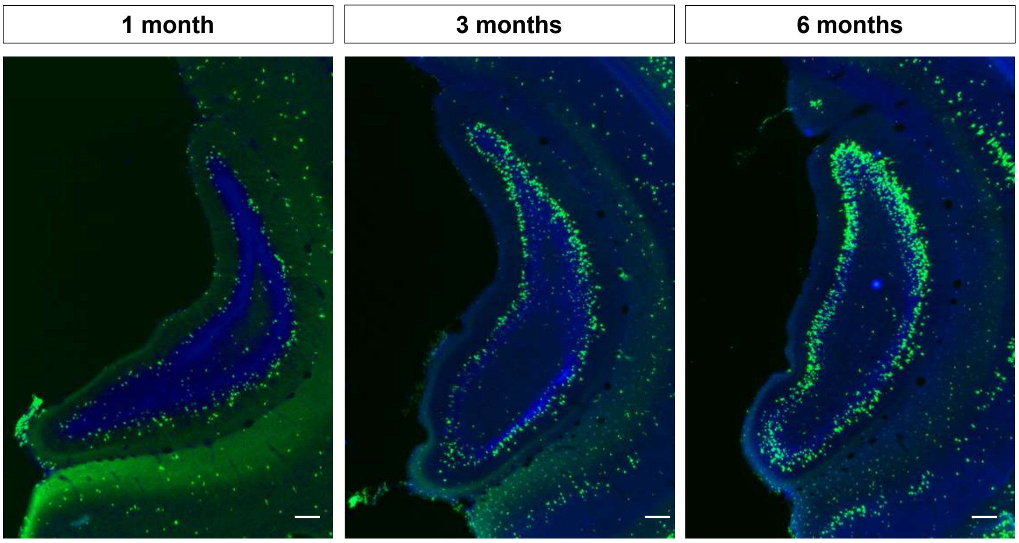

Figure S5

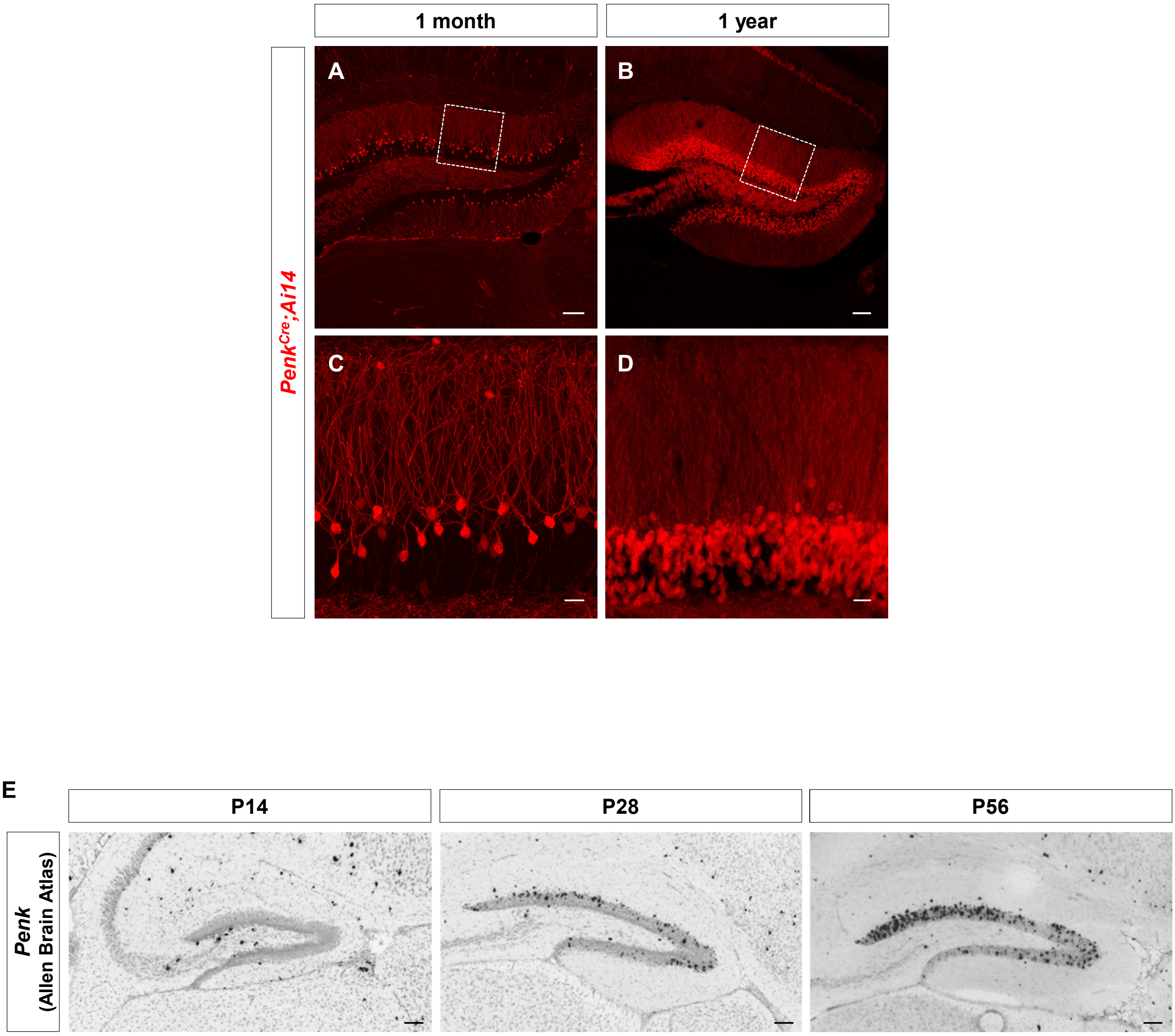

1 month
1 year
A
B
C
D
$Penk^{Cre}$;Ai14
E
P14
P28
P56
Penk
(Allen Brain Atlas)
